# *trans*-{1,8-Bis[(*R*)-α-methyl­benz­yl]-1,3,6,8,10,13-hexa­azacyclo­tetra­deca­ne}dithio­cyanato­nickel(II)

**DOI:** 10.1107/S1600536809002700

**Published:** 2009-01-28

**Authors:** Jong Won Shin, Kil Sik Min

**Affiliations:** a Department of Chemistry, Kyungpook National University, Daegu 702-701, Republic of Korea; b Department of Chemistry Education, Kyungpook National University, Daegu 702-701, Republic of Korea

## Abstract

The title compound, [Ni(NCS)_2_(C_24_H_38_N_6_)], is a thio­cyanate-coordinated aza­macrocyclic nickel(II) complex. There are two independent mol­ecules in the asymmetric unit and their bond lengths and angles are similar. Both Ni atoms have a tetra­gonally distorted octa­hedral geometry, in which the Ni^II^ ion is coordinated by the four secondary N atoms of the aza­macrocyclic ligand and by two N atoms of the thio­cyanate ions. The average equatorial Ni—N bond lengths are shorter than the average axial Ni—N bond lengths [2.071 (1) and 2.115 (2) Å, respectively]. N—H⋯S hydrogen-bonding inter­actions between a secondary amine N atom and the adjacent thio­cyanate ion leads to a polymeric chain along [100].

## Related literature

For general background, see: Banerjee & Zubieta (2004 ▶); Du *et al.* (2003 ▶); Gao *et al.* (2005 ▶); Han *et al.* (2008 ▶); Katsuki *et al.* (2000 ▶); Lehn (1995 ▶); Leonard *et al.* (2007 ▶); Stølevik & Postmyr (1997 ▶).
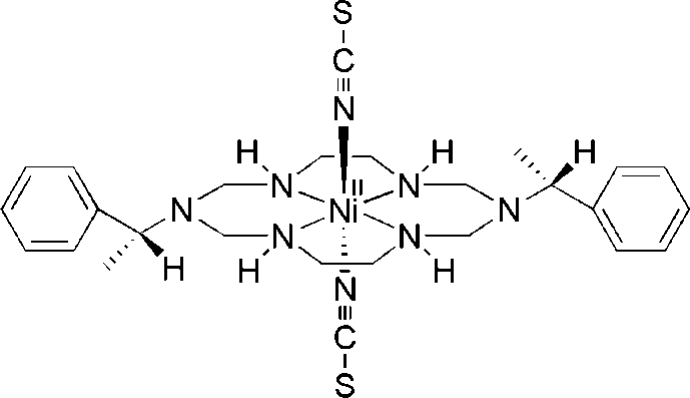


## Experimental

### 

#### Crystal data

[Ni(NCS)_2_(C_24_H_38_N_6_)]
*M* *_r_* = 585.47
Orthorhombic, 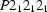

*a* = 8.5313 (5) Å
*b* = 15.3141 (10) Å
*c* = 44.004 (3) Å
*V* = 5749.1 (6) Å^3^
*Z* = 8
Mo *K*α radiation
μ = 0.85 mm^−1^
*T* = 173 (2) K
0.36 × 0.17 × 0.16 mm

#### Data collection

Siemens SMART CCD diffractometer
Absorption correction: multi-scan (*SADABS*; Sheldrick, 1996 ▶) *T*_min_ = 0.679, *T*_max_ = 0.873
43415 measured reflections
14300 independent reflections
8093 reflections with *I* > 2σ(*I*)
*R*_int_ = 0.103

#### Refinement

*R*[*F* ^2^ > 2σ(*F* ^2^)] = 0.057
*wR*(*F* ^2^) = 0.149
*S* = 1.04
14300 reflections
668 parameters
H-atom parameters constrained
Δρ_max_ = 0.81 e Å^−3^
Δρ_min_ = −0.93 e Å^−3^
Absolute structure: Flack (1983 ▶)
Flack parameter: −0.004 (17)

### 

Data collection: *SMART* (Siemens, 1996 ▶); cell refinement: *SAINT* (Siemens, 1996 ▶); data reduction: *SHELXTL* (Sheldrick, 2008 ▶); program(s) used to solve structure: *SHELXS97* (Sheldrick, 2008 ▶); program(s) used to refine structure: *SHELXL97* (Sheldrick, 2008 ▶); molecular graphics: *ORTEP-3* (Farrugia, 1997 ▶); software used to prepare material for publication: *SHELXL97*.

## Supplementary Material

Crystal structure: contains datablocks global, I. DOI: 10.1107/S1600536809002700/ez2157sup1.cif

Structure factors: contains datablocks I. DOI: 10.1107/S1600536809002700/ez2157Isup2.hkl

Additional supplementary materials:  crystallographic information; 3D view; checkCIF report

## Figures and Tables

**Table 1:** Hydrogen-bond geometry (Å, °)

*D*—H⋯*A*	*D*—H	H⋯*A*	*D*⋯*A*	*D*—H⋯*A*
N3—H3⋯S1^i^	0.93	2.82	3.439 (4)	125
N6—H6⋯S2^ii^	0.93	2.71	3.347 (4)	127
N11—H11⋯S3^ii^	0.93	2.89	3.614 (5)	136
N14—H14⋯S4^i^	0.93	2.66	3.418 (4)	139

Symmetry codes: (i) 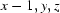; (ii) 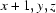.

**Fig. 1.**
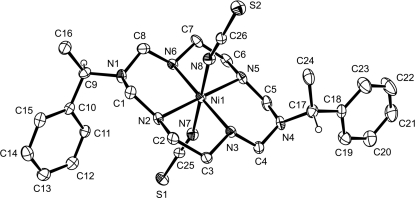
ORTEP drawing of the molecular title compound with atomic numbering scheme and ellipsoids at 40% probability.

**Fig. 2.**
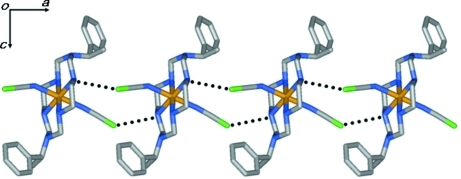
Perspective view of the title compound showing a one-dimensional chain formed by N—H···S hydrogen bonding interactions.

## supplementary crystallographic information

### Comment

Chiral complexes have attracted considerable attention in chemistry and material science because of their potential applications for molecular recognition, catalysis, and separation (Lehn, 1995; Katsuki *et al.*, 2000). Very recently lanthanide metal complexes with chiral ligands have been studied in the self-assembly of luminescent materials (Leonard *et al.*, 2007). However, the study of chiral macrocyclic metal complexes has been limited due to the difficulty of preparation, although these complexes can be utilized as chiral building blocks (Du *et al.*, 2003; Gao *et al.*, 2005). Here, we report the synthesis and crystal structure of the nickel(II) azamacrocyclic chiral complex, *trans*-dithiocyanato(1,8-di(*R*-*α*-methylbenzyl)- 1,3,6,8,10,13-hexaazacyclotetradecane)nickel(II), with two thiocyanate ions axially.

In the title compound, the coordination geometry around the nickel(II) ion is a tetragonally distorted octahedron in which the nickel(II) ion is coordinated to the four secondary N atoms of the azamacrocyclic ligand in a square-planar fashion and two N atoms from the thiocyanate ions at the axial positions as shown in Figure 1. The average Ni—N_eq_ and Ni—N_ax_ bond distances are 2.071 (1) and 2.115 (2) Å, respectively. The former is slightly less than the latter, which can be attributed to the Jahn-Teller distortion of the nickel(II) ion and/or the ring contraction of the azamacrocyclic ligand. In the coordinated thiocyanate ions, the average N—C and C—S bond distances are 1.166 (3) and 1.621 (3) Å, respectively. The former is very similar to a CN triple bond length, while the latter is slightly shorter than the normal CS single bond distance (Stølevik & Postmyr, 1997; Banerjee & Zubieta, 2004). The pendant arms of the azamacrocyclic ligand have chiral carbon atoms (*R* type). All thiocyanate ions binding nickel(II) ions axially are involved in N—H···S hydrogen bonding interactions (Table 1), which give rise to one-dimensional polymeric chains propagating along the *a* axis (Figure 2). The shortest Ni···Ni intrachain separation within the hydrogen-bonded one-dimensional polymer is 8.531 (1) Å and is about 5% longer than the shortest interchain Ni···Ni distance of 8.166 (1) Å.

### Experimental

The title compound is prepared as follows: To an MeCN solution (10 ml) of [Ni(C_24_H_38_N_6_)](ClO_4_)_2_ (0.10 g, 0.15 mmol) (Han *et al.*, 2008) was added dropwise an aqueous solution (10 ml) containing NaSCN (0.024 g, 0.30 mmol) at ambient temperature. The color of the solution changed from yellow to pale pink. The mixture was stirred for 30 min during which time a pink precipitate formed which was collected by filtration, washed with MeCN and water, and dried in air. Single crystals of the title compound suitable for X-ray crystallography were grown by layering of the MeCN solution of [Ni(C_24_H_38_N_6_)](ClO_4_)_2_ on the aqueous solution of NaSCN within one week.

### Refinement

All H atoms in the title compound were placed in geometrically idealized positions and constrained to ride on their parent atoms, with C—H distances of 0.95 (ring H atoms) or 0.99–1.00 (open chain H atoms) Å and N—H distance of 0.93 Å, and with *U*_iso_(H) values of 1.2 times the equivalent anisotropic displacement parameters of the parent C and N atoms.

### Crystal data

**Table:**

[Ni(NCS)_2_(C_24_H_38_N_6_)]	*F*(000) = 2480
*M**_r_* = 585.47	*D*_x_ = 1.353 Mg m^−^^3^
Orthorhombic, *P*2_1_2_1_2_1_	Mo *K*α radiation, λ = 0.71073 Å
Hall symbol: P 2ac 2ab	Cell parameters from 6933 reflections
*a* = 8.5313 (5) Å	θ = 2.6–22.8°
*b* = 15.3141 (10) Å	µ = 0.85 mm^−^^1^
*c* = 44.004 (3) Å	*T* = 173 K
*V* = 5749.1 (6) Å^3^	Block, violet
*Z* = 8	0.36 × 0.17 × 0.16 mm

### Data collection

**Table:**

Siemens SMART CCD diffractometer	14300 independent reflections
Radiation source: fine-focus sealed tube	8093 reflections with *I* > 2σ(*I*)
graphite	*R*_int_ = 0.103
φ and ω scans	θ_max_ = 28.4°, θ_min_ = 0.9°
Absorption correction: multi-scan (*SADABS*; Sheldrick, 1996)	*h* = −11→10
*T*_min_ = 0.679, *T*_max_ = 0.873	*k* = −20→20
43415 measured reflections	*l* = −58→43

### Refinement

**Table:**

Refinement on *F*^2^	Secondary atom site location: difference Fourier map
Least-squares matrix: full	Hydrogen site location: inferred from neighbouring sites
*R*[*F*^2^ > 2σ(*F*^2^)] = 0.057	H-atom parameters constrained
*wR*(*F*^2^) = 0.149	*w* = 1/[σ^2^(*F*_o_^2^) + (0.0358*P*)^2^ + 2.5654*P*] where *P* = (*F*_o_^2^ + 2*F*_c_^2^)/3
*S* = 1.04	(Δ/σ)_max_ = 0.001
14300 reflections	Δρ_max_ = 0.81 e Å^−^^3^
668 parameters	Δρ_min_ = −0.93 e Å^−^^3^
0 restraints	Absolute structure: Flack (1983)
Primary atom site location: structure-invariant direct methods	Flack parameter: −0.004 (17)

### Special details

**Table:**

Geometry. All e.s.d.'s (except the e.s.d. in the dihedral angle between two l.s. planes) are estimated using the full covariance matrix. The cell e.s.d.'s are taken into account individually in the estimation of e.s.d.'s in distances, angles and torsion angles; correlations between e.s.d.'s in cell parameters are only used when they are defined by crystal symmetry. An approximate (isotropic) treatment of cell e.s.d.'s is used for estimating e.s.d.'s involving l.s. planes.
Refinement. Refinement of *F*^2^ against ALL reflections. The weighted *R*-factor *wR* and goodness of fit *S* are based on *F*^2^, conventional *R*-factors *R* are based on *F*, with *F* set to zero for negative *F*^2^. The threshold expression of *F*^2^ > σ(*F*^2^) is used only for calculating *R*-factors(gt) *etc*. and is not relevant to the choice of reflections for refinement. *R*-factors based on *F*^2^ are statistically about twice as large as those based on *F*, and *R*- factors based on ALL data will be even larger.

### Fractional atomic coordinates and isotropic or equivalent isotropic displacement parameters (Å^2^)

**Table:**

	*x*	*y*	*z*	*U*_iso_*/*U*_eq_	
Ni1	−0.42128 (7)	−0.26127 (4)	−0.137939 (15)	0.02628 (15)	
N1	−0.2552 (5)	−0.2772 (3)	−0.20285 (10)	0.0322 (11)	
N2	−0.4025 (5)	−0.3641 (3)	−0.16856 (10)	0.0304 (10)	
H2	−0.3118	−0.3948	−0.1638	0.037*	
N3	−0.5759 (5)	−0.3415 (2)	−0.11490 (10)	0.0282 (10)	
H3	−0.6770	−0.3229	−0.1194	0.034*	
N4	−0.5705 (5)	−0.2512 (3)	−0.06892 (10)	0.0365 (10)	
N5	−0.4383 (5)	−0.1595 (3)	−0.10661 (10)	0.0335 (11)	
H5	−0.5298	−0.1288	−0.1109	0.040*	
N6	−0.2683 (5)	−0.1803 (3)	−0.16088 (10)	0.0280 (10)	
H6	−0.1672	−0.1980	−0.1559	0.034*	
N7	−0.2264 (5)	−0.3164 (3)	−0.11474 (11)	0.0375 (12)	
N8	−0.6143 (5)	−0.2084 (3)	−0.16108 (11)	0.0349 (11)	
S1	0.04603 (16)	−0.41163 (10)	−0.10308 (4)	0.0439 (4)	
S2	−0.89623 (17)	−0.13257 (10)	−0.17838 (4)	0.0454 (4)	
C1	−0.3884 (6)	−0.3352 (3)	−0.20038 (12)	0.0312 (13)	
H1A	−0.3736	−0.3863	−0.2138	0.037*	
H1B	−0.4851	−0.3044	−0.2067	0.037*	
C2	−0.5377 (6)	−0.4218 (3)	−0.16175 (12)	0.0319 (13)	
H2A	−0.5197	−0.4804	−0.1706	0.038*	
H2B	−0.6343	−0.3973	−0.1708	0.038*	
C3	−0.5565 (6)	−0.4290 (3)	−0.12744 (13)	0.0352 (14)	
H3A	−0.6493	−0.4652	−0.1225	0.042*	
H3B	−0.4628	−0.4572	−0.1185	0.042*	
C4	−0.5554 (7)	−0.3370 (4)	−0.08118 (12)	0.0359 (14)	
H4A	−0.6343	−0.3753	−0.0715	0.043*	
H4B	−0.4504	−0.3600	−0.0759	0.043*	
C5	−0.4477 (7)	−0.1893 (4)	−0.07469 (13)	0.0402 (15)	
H5A	−0.3462	−0.2160	−0.0690	0.048*	
H5B	−0.4639	−0.1378	−0.0615	0.048*	
C6	−0.3048 (6)	−0.1014 (3)	−0.11345 (14)	0.0364 (14)	
H6A	−0.2068	−0.1262	−0.1050	0.044*	
H6B	−0.3220	−0.0432	−0.1042	0.044*	
C7	−0.2918 (6)	−0.0930 (4)	−0.14761 (14)	0.0394 (15)	
H7A	−0.3886	−0.0665	−0.1559	0.047*	
H7B	−0.2024	−0.0547	−0.1529	0.047*	
C8	−0.2837 (6)	−0.1871 (4)	−0.19439 (14)	0.0367 (14)	
H8A	−0.3902	−0.1692	−0.2008	0.044*	
H8B	−0.2067	−0.1484	−0.2045	0.044*	
C9	−0.1478 (6)	−0.2927 (4)	−0.22812 (13)	0.0335 (13)	
H9A	−0.0605	−0.2495	−0.2261	0.040*	
C10	−0.0759 (6)	−0.3830 (3)	−0.22447 (12)	0.0306 (12)	
C11	0.0020 (6)	−0.4024 (4)	−0.19683 (13)	0.0341 (13)	
H11A	0.0072	−0.3594	−0.1813	0.041*	
C12	0.0705 (7)	−0.4831 (4)	−0.19227 (14)	0.0420 (14)	
H12A	0.1230	−0.4953	−0.1737	0.050*	
C13	0.0628 (7)	−0.5459 (4)	−0.21461 (15)	0.0455 (16)	
H13A	0.1101	−0.6013	−0.2114	0.055*	
C14	−0.0131 (7)	−0.5287 (4)	−0.24141 (15)	0.0467 (17)	
H14A	−0.0177	−0.5725	−0.2567	0.056*	
C15	−0.0835 (7)	−0.4479 (4)	−0.24657 (13)	0.0383 (13)	
H15A	−0.1367	−0.4372	−0.2652	0.046*	
C16	−0.2242 (6)	−0.2766 (4)	−0.25908 (13)	0.0438 (16)	
H16A	−0.2742	−0.2190	−0.2592	0.066*	
H16B	−0.3034	−0.3217	−0.2629	0.066*	
H16C	−0.1440	−0.2790	−0.2750	0.066*	
C17	−0.7257 (6)	−0.2194 (4)	−0.05969 (13)	0.0384 (14)	
H17A	−0.7910	−0.2728	−0.0564	0.046*	
C18	−0.7153 (6)	−0.1745 (4)	−0.02895 (13)	0.0363 (14)	
C19	−0.6286 (7)	−0.2120 (4)	−0.00594 (14)	0.0474 (17)	
H19A	−0.5739	−0.2649	−0.0097	0.057*	
C20	−0.6200 (8)	−0.1737 (5)	0.02239 (15)	0.0557 (18)	
H20A	−0.5632	−0.2018	0.0382	0.067*	
C21	−0.6924 (9)	−0.0955 (5)	0.02797 (17)	0.068 (2)	
H21A	−0.6834	−0.0679	0.0472	0.082*	
C22	−0.7772 (11)	−0.0585 (6)	0.00542 (19)	0.086 (3)	
H22A	−0.8288	−0.0045	0.0090	0.104*	
C23	−0.7900 (9)	−0.0976 (5)	−0.02252 (16)	0.065 (2)	
H23	−0.8523	−0.0707	−0.0378	0.078*	
C24	−0.8099 (7)	−0.1669 (4)	−0.08374 (14)	0.0486 (17)	
H24A	−0.8077	−0.1988	−0.1031	0.073*	
H24B	−0.7578	−0.1104	−0.0862	0.073*	
H24C	−0.9189	−0.1576	−0.0775	0.073*	
C25	−0.1125 (6)	−0.3557 (3)	−0.11025 (12)	0.0292 (12)	
C26	−0.7334 (6)	−0.1772 (3)	−0.16823 (12)	0.0286 (12)	
Ni2	−0.09613 (7)	−0.75727 (4)	−0.112058 (15)	0.02774 (16)	
N9	−0.1996 (6)	−0.7839 (3)	−0.18264 (10)	0.0353 (11)	
N10	−0.0978 (5)	−0.8651 (3)	−0.14084 (10)	0.0315 (10)	
H10	−0.1938	−0.8931	−0.1385	0.038*	
N11	0.0348 (5)	−0.8330 (3)	−0.08278 (10)	0.0340 (11)	
H11	0.1397	−0.8187	−0.0857	0.041*	
N12	0.0131 (5)	−0.7279 (3)	−0.04078 (11)	0.0395 (12)	
N13	−0.1034 (5)	−0.6485 (3)	−0.08377 (10)	0.0362 (11)	
H13	−0.0121	−0.6166	−0.0870	0.043*	
N14	−0.2280 (5)	−0.6848 (3)	−0.14172 (10)	0.0286 (10)	
H14	−0.3312	−0.7040	−0.1402	0.034*	
N15	−0.3111 (5)	−0.8075 (3)	−0.09316 (12)	0.0441 (13)	
N16	0.1144 (5)	−0.7100 (3)	−0.13003 (11)	0.0363 (11)	
S3	−0.57566 (18)	−0.91335 (11)	−0.09892 (4)	0.0505 (4)	
S4	0.38600 (18)	−0.66232 (11)	−0.16098 (4)	0.0537 (5)	
C27	−0.0798 (6)	−0.8436 (4)	−0.17323 (12)	0.0342 (13)	
H27A	0.0245	−0.8171	−0.1767	0.041*	
H27B	−0.0864	−0.8977	−0.1855	0.041*	
C28	0.0249 (6)	−0.9231 (4)	−0.12818 (14)	0.0388 (15)	
H28A	0.1298	−0.9019	−0.1344	0.047*	
H28B	0.0111	−0.9831	−0.1361	0.047*	
C29	0.0124 (6)	−0.9235 (3)	−0.09346 (14)	0.0388 (15)	
H29A	−0.0919	−0.9454	−0.0871	0.047*	
H29B	0.0936	−0.9621	−0.0846	0.047*	
C30	−0.0025 (7)	−0.8179 (4)	−0.05011 (13)	0.0423 (15)	
H30A	−0.1114	−0.8373	−0.0462	0.051*	
H30B	0.0680	−0.8544	−0.0375	0.051*	
C31	−0.1116 (7)	−0.6700 (4)	−0.05131 (12)	0.0398 (14)	
H31A	−0.1073	−0.6151	−0.0395	0.048*	
H31B	−0.2139	−0.6980	−0.0471	0.048*	
C32	−0.2367 (6)	−0.5964 (4)	−0.09557 (14)	0.0399 (15)	
H32A	−0.2327	−0.5362	−0.0874	0.048*	
H32B	−0.3375	−0.6233	−0.0895	0.048*	
C33	−0.2220 (6)	−0.5949 (4)	−0.13008 (13)	0.0355 (14)	
H33A	−0.3086	−0.5602	−0.1389	0.043*	
H33B	−0.1216	−0.5673	−0.1360	0.043*	
C34	−0.1791 (7)	−0.6952 (4)	−0.17348 (13)	0.0369 (14)	
H34A	−0.2426	−0.6565	−0.1867	0.044*	
H34B	−0.0676	−0.6783	−0.1757	0.044*	
C35	−0.2490 (6)	−0.8016 (4)	−0.21473 (13)	0.0380 (14)	
H35	−0.1526	−0.8155	−0.2267	0.046*	
C36	−0.3563 (6)	−0.8813 (3)	−0.21594 (13)	0.0309 (13)	
C37	−0.3497 (6)	−0.9361 (4)	−0.24072 (13)	0.0365 (14)	
H37	−0.2736	−0.9264	−0.2561	0.044*	
C38	−0.4543 (7)	−1.0059 (4)	−0.24333 (14)	0.0435 (16)	
H38	−0.4500	−1.0433	−0.2605	0.052*	
C39	−0.5631 (7)	−1.0203 (4)	−0.22100 (15)	0.0439 (15)	
H39	−0.6340	−1.0679	−0.2228	0.053*	
C40	−0.5708 (7)	−0.9666 (3)	−0.19603 (14)	0.0398 (14)	
H40	−0.6474	−0.9768	−0.1808	0.048*	
C41	−0.4667 (6)	−0.8977 (4)	−0.19312 (13)	0.0374 (14)	
H41	−0.4703	−0.8615	−0.1756	0.045*	
C42	−0.3315 (7)	−0.7254 (4)	−0.23042 (14)	0.0487 (17)	
H42A	−0.2607	−0.6751	−0.2312	0.073*	
H42B	−0.4260	−0.7097	−0.2190	0.073*	
H42C	−0.3609	−0.7425	−0.2511	0.073*	
C43	0.1719 (6)	−0.6900 (4)	−0.04258 (14)	0.0384 (15)	
H43	0.1953	−0.6751	−0.0642	0.046*	
C44	0.1845 (7)	−0.6076 (4)	−0.02341 (13)	0.0386 (14)	
C45	0.2708 (9)	−0.5376 (5)	−0.03401 (16)	0.064 (2)	
H45	0.3126	−0.5389	−0.0540	0.077*	
C46	0.2966 (11)	−0.4656 (5)	−0.01568 (19)	0.086 (3)	
H46	0.3594	−0.4189	−0.0230	0.103*	
C47	0.2330 (6)	−0.4605 (3)	0.01289 (11)	0.063 (2)	
H47	0.2497	−0.4107	0.0254	0.075*	
C48	0.1452 (6)	−0.5289 (3)	0.02279 (11)	0.0493 (17)	
H48	0.0983	−0.5258	0.0423	0.059*	
C49	0.1221 (7)	−0.6025 (4)	0.00538 (14)	0.0467 (16)	
H49	0.0629	−0.6499	0.0133	0.056*	
C50	0.2954 (7)	−0.7538 (5)	−0.03109 (15)	0.0561 (18)	
H50A	0.2973	−0.8054	−0.0442	0.084*	
H50B	0.2699	−0.7714	−0.0103	0.084*	
H50C	0.3985	−0.7255	−0.0314	0.084*	
C51	−0.4230 (7)	−0.8509 (4)	−0.09556 (13)	0.0372 (13)	
C52	0.2301 (6)	−0.6896 (3)	−0.14247 (13)	0.0314 (13)	

### Atomic displacement parameters (Å^2^)

**Table:**

	*U*^11^	*U*^22^	*U*^33^	*U*^12^	*U*^13^	*U*^23^
Ni1	0.0199 (3)	0.0276 (3)	0.0314 (4)	0.0002 (3)	−0.0008 (3)	−0.0027 (3)
N1	0.025 (2)	0.038 (3)	0.034 (3)	−0.008 (2)	0.006 (2)	−0.009 (2)
N2	0.026 (2)	0.033 (2)	0.032 (3)	0.003 (2)	0.003 (2)	−0.005 (2)
N3	0.023 (2)	0.028 (2)	0.034 (3)	−0.0013 (19)	0.003 (2)	−0.0027 (19)
N4	0.029 (2)	0.045 (3)	0.035 (3)	0.007 (2)	0.006 (2)	−0.011 (2)
N5	0.026 (2)	0.031 (2)	0.043 (3)	0.002 (2)	0.003 (2)	−0.010 (2)
N6	0.020 (2)	0.026 (2)	0.038 (3)	−0.0015 (18)	0.003 (2)	0.000 (2)
N7	0.028 (2)	0.045 (3)	0.039 (3)	0.004 (2)	−0.003 (2)	−0.002 (2)
N8	0.029 (2)	0.036 (3)	0.039 (3)	−0.003 (2)	0.000 (2)	0.004 (2)
S1	0.0275 (7)	0.0465 (9)	0.0578 (11)	0.0074 (6)	−0.0065 (7)	−0.0032 (8)
S2	0.0241 (7)	0.0555 (10)	0.0565 (11)	0.0053 (7)	−0.0048 (7)	0.0050 (8)
C1	0.028 (3)	0.035 (3)	0.031 (3)	−0.012 (2)	0.001 (3)	−0.006 (2)
C2	0.037 (3)	0.023 (3)	0.035 (3)	−0.010 (2)	0.001 (3)	−0.009 (2)
C3	0.036 (3)	0.023 (3)	0.046 (4)	−0.004 (2)	0.014 (3)	0.000 (2)
C4	0.037 (3)	0.041 (3)	0.030 (3)	0.008 (3)	0.003 (3)	−0.002 (3)
C5	0.034 (3)	0.049 (4)	0.037 (4)	−0.002 (3)	0.001 (3)	−0.021 (3)
C6	0.027 (3)	0.025 (3)	0.057 (4)	−0.008 (2)	−0.006 (3)	−0.021 (3)
C7	0.030 (3)	0.025 (3)	0.064 (4)	−0.002 (2)	0.007 (3)	−0.010 (3)
C8	0.027 (3)	0.036 (3)	0.047 (4)	0.001 (2)	0.002 (3)	0.011 (3)
C9	0.029 (3)	0.039 (3)	0.033 (3)	−0.002 (2)	−0.002 (3)	0.007 (3)
C10	0.026 (3)	0.042 (3)	0.024 (3)	−0.004 (3)	0.000 (3)	0.002 (2)
C11	0.034 (3)	0.041 (3)	0.027 (3)	−0.002 (3)	−0.001 (3)	−0.003 (3)
C12	0.035 (3)	0.050 (4)	0.041 (4)	−0.003 (3)	−0.001 (3)	0.003 (3)
C13	0.039 (3)	0.041 (3)	0.056 (4)	−0.004 (3)	0.011 (3)	0.006 (3)
C14	0.044 (4)	0.046 (4)	0.050 (5)	−0.003 (3)	0.004 (3)	−0.013 (3)
C15	0.030 (3)	0.052 (4)	0.032 (3)	−0.010 (3)	0.000 (3)	−0.006 (3)
C16	0.035 (3)	0.059 (4)	0.037 (4)	0.011 (3)	0.001 (3)	0.006 (3)
C17	0.029 (3)	0.049 (4)	0.036 (3)	0.002 (3)	0.000 (3)	0.001 (3)
C18	0.035 (3)	0.041 (3)	0.033 (3)	−0.002 (3)	0.009 (3)	−0.008 (3)
C19	0.056 (4)	0.048 (4)	0.038 (4)	0.009 (3)	−0.008 (3)	−0.010 (3)
C20	0.050 (4)	0.077 (5)	0.040 (4)	0.005 (4)	−0.010 (3)	−0.007 (4)
C21	0.078 (5)	0.084 (6)	0.041 (4)	0.005 (5)	0.005 (4)	−0.024 (4)
C22	0.114 (7)	0.086 (6)	0.058 (5)	0.055 (6)	−0.011 (5)	−0.033 (5)
C23	0.079 (5)	0.075 (5)	0.040 (4)	0.033 (4)	−0.013 (4)	−0.017 (4)
C24	0.042 (3)	0.068 (5)	0.035 (4)	0.015 (3)	−0.004 (3)	−0.015 (3)
C25	0.028 (3)	0.027 (3)	0.032 (3)	−0.002 (2)	−0.005 (3)	0.000 (2)
C26	0.028 (3)	0.028 (3)	0.030 (3)	−0.003 (2)	0.003 (2)	−0.002 (2)
Ni2	0.0234 (3)	0.0315 (4)	0.0284 (4)	−0.0006 (3)	−0.0012 (3)	−0.0009 (3)
N9	0.049 (3)	0.034 (3)	0.023 (3)	0.002 (2)	0.001 (2)	−0.001 (2)
N10	0.029 (2)	0.029 (2)	0.037 (3)	0.000 (2)	−0.005 (2)	−0.001 (2)
N11	0.024 (2)	0.037 (3)	0.041 (3)	−0.0031 (19)	−0.010 (2)	−0.001 (2)
N12	0.038 (3)	0.042 (3)	0.038 (3)	0.004 (2)	−0.004 (2)	−0.009 (2)
N13	0.032 (2)	0.037 (3)	0.040 (3)	0.005 (2)	−0.006 (2)	−0.005 (2)
N14	0.027 (2)	0.026 (2)	0.033 (3)	0.0014 (18)	−0.007 (2)	0.001 (2)
N15	0.030 (3)	0.059 (3)	0.043 (3)	−0.008 (2)	0.004 (2)	0.001 (3)
N16	0.031 (2)	0.039 (3)	0.039 (3)	−0.005 (2)	0.002 (2)	0.000 (2)
S3	0.0303 (8)	0.0555 (10)	0.0658 (12)	−0.0068 (8)	−0.0041 (8)	−0.0032 (9)
S4	0.0299 (8)	0.0550 (10)	0.0761 (13)	−0.0029 (7)	0.0103 (8)	0.0162 (9)
C27	0.029 (3)	0.042 (3)	0.032 (3)	0.005 (3)	−0.004 (3)	−0.005 (3)
C28	0.034 (3)	0.028 (3)	0.054 (4)	−0.001 (2)	−0.006 (3)	−0.009 (3)
C29	0.034 (3)	0.028 (3)	0.054 (4)	−0.002 (2)	−0.010 (3)	0.004 (3)
C30	0.040 (3)	0.053 (4)	0.034 (4)	−0.004 (3)	0.001 (3)	0.010 (3)
C31	0.040 (3)	0.051 (4)	0.029 (3)	0.002 (3)	0.001 (3)	−0.005 (3)
C32	0.033 (3)	0.039 (3)	0.048 (4)	0.012 (3)	−0.008 (3)	−0.014 (3)
C33	0.034 (3)	0.035 (3)	0.037 (4)	0.004 (3)	−0.005 (3)	−0.001 (3)
C34	0.035 (3)	0.047 (4)	0.029 (3)	−0.005 (3)	−0.006 (3)	0.000 (3)
C35	0.036 (3)	0.052 (4)	0.026 (3)	−0.001 (3)	−0.004 (3)	−0.003 (3)
C36	0.028 (3)	0.033 (3)	0.032 (3)	0.000 (2)	−0.007 (3)	0.005 (3)
C37	0.034 (3)	0.045 (4)	0.031 (3)	0.001 (3)	−0.003 (3)	−0.003 (3)
C38	0.052 (4)	0.037 (3)	0.041 (4)	0.009 (3)	−0.016 (3)	−0.004 (3)
C39	0.040 (3)	0.035 (3)	0.057 (4)	0.001 (3)	−0.013 (3)	0.003 (3)
C40	0.035 (3)	0.038 (3)	0.046 (4)	−0.008 (3)	−0.001 (3)	0.010 (3)
C41	0.040 (3)	0.040 (3)	0.032 (3)	0.008 (3)	0.003 (3)	0.003 (3)
C42	0.061 (4)	0.048 (4)	0.036 (4)	−0.019 (3)	−0.017 (3)	0.010 (3)
C43	0.034 (3)	0.056 (4)	0.024 (3)	−0.002 (3)	−0.003 (3)	−0.005 (3)
C44	0.040 (3)	0.048 (4)	0.028 (3)	0.005 (3)	−0.005 (3)	−0.004 (3)
C45	0.099 (6)	0.057 (5)	0.036 (4)	−0.020 (4)	0.021 (4)	−0.002 (4)
C46	0.149 (8)	0.053 (5)	0.057 (5)	−0.039 (5)	0.031 (6)	0.000 (4)
C47	0.087 (6)	0.050 (4)	0.051 (5)	−0.006 (4)	0.015 (4)	−0.015 (4)
C48	0.059 (4)	0.055 (4)	0.034 (4)	−0.006 (3)	0.004 (3)	−0.005 (3)
C49	0.048 (4)	0.055 (4)	0.037 (4)	−0.014 (3)	0.002 (3)	−0.004 (3)
C50	0.046 (3)	0.055 (4)	0.067 (5)	0.011 (4)	−0.019 (3)	−0.014 (4)
C51	0.030 (3)	0.047 (3)	0.035 (3)	0.002 (3)	0.000 (3)	0.003 (3)
C52	0.028 (3)	0.025 (3)	0.042 (4)	−0.001 (2)	−0.003 (3)	−0.001 (3)

### Geometric parameters (Å, °)

**Table:**

Ni1—N6	2.064 (4)	Ni2—N14	2.049 (4)
Ni1—N3	2.068 (4)	Ni2—N11	2.063 (4)
Ni1—N2	2.079 (4)	Ni2—N13	2.080 (4)
Ni1—N5	2.086 (4)	Ni2—N10	2.081 (4)
Ni1—N8	2.098 (5)	Ni2—N16	2.092 (4)
Ni1—N7	2.126 (5)	Ni2—N15	2.156 (5)
N1—C1	1.446 (6)	N9—C34	1.429 (7)
N1—C8	1.449 (7)	N9—C27	1.432 (7)
N1—C9	1.460 (7)	N9—C35	1.498 (7)
N2—C1	1.473 (6)	N10—C27	1.471 (7)
N2—C2	1.484 (6)	N10—C28	1.482 (7)
N2—H2	0.9300	N10—H10	0.9300
N3—C3	1.459 (6)	N11—C29	1.476 (7)
N3—C4	1.496 (6)	N11—C30	1.491 (7)
N3—H3	0.9300	N11—H11	0.9300
N4—C4	1.426 (7)	N12—C30	1.446 (7)
N4—C5	1.436 (7)	N12—C31	1.460 (7)
N4—C17	1.468 (6)	N12—C43	1.475 (7)
N5—C6	1.477 (6)	N13—C31	1.467 (7)
N5—C5	1.479 (7)	N13—C32	1.484 (7)
N5—H5	0.9300	N13—H13	0.9300
N6—C7	1.473 (7)	N14—C34	1.467 (7)
N6—C8	1.484 (7)	N14—C33	1.470 (7)
N6—H6	0.9300	N14—H14	0.9300
N7—C25	1.160 (6)	N15—C51	1.168 (7)
N8—C26	1.167 (6)	N16—C52	1.171 (6)
S1—C25	1.632 (5)	S3—C51	1.623 (6)
S2—C26	1.611 (6)	S4—C52	1.615 (6)
C1—H1A	0.9900	C27—H27A	0.9900
C1—H1B	0.9900	C27—H27B	0.9900
C2—C3	1.522 (7)	C28—C29	1.531 (8)
C2—H2A	0.9900	C28—H28A	0.9900
C2—H2B	0.9900	C28—H28B	0.9900
C3—H3A	0.9900	C29—H29A	0.9900
C3—H3B	0.9900	C29—H29B	0.9900
C4—H4A	0.9900	C30—H30A	0.9900
C4—H4B	0.9900	C30—H30B	0.9900
C5—H5A	0.9900	C31—H31A	0.9900
C5—H5B	0.9900	C31—H31B	0.9900
C6—C7	1.513 (8)	C32—C33	1.524 (8)
C6—H6A	0.9900	C32—H32A	0.9900
C6—H6B	0.9900	C32—H32B	0.9900
C7—H7A	0.9900	C33—H33A	0.9900
C7—H7B	0.9900	C33—H33B	0.9900
C8—H8A	0.9900	C34—H34A	0.9900
C8—H8B	0.9900	C34—H34B	0.9900
C9—C10	1.521 (8)	C35—C36	1.527 (8)
C9—C16	1.530 (7)	C35—C42	1.528 (8)
C9—H9A	1.0000	C35—H35	1.0000
C10—C15	1.393 (7)	C36—C37	1.377 (8)
C10—C11	1.418 (7)	C36—C41	1.399 (7)
C11—C12	1.382 (8)	C37—C38	1.397 (8)
C11—H11A	0.9500	C37—H37	0.9500
C12—C13	1.377 (8)	C38—C39	1.370 (8)
C12—H12A	0.9500	C38—H38	0.9500
C13—C14	1.370 (9)	C39—C40	1.374 (8)
C13—H13A	0.9500	C39—H39	0.9500
C14—C15	1.394 (8)	C40—C41	1.385 (7)
C14—H14A	0.9500	C40—H40	0.9500
C15—H15A	0.9500	C41—H41	0.9500
C16—H16A	0.9800	C42—H42A	0.9800
C16—H16B	0.9800	C42—H42B	0.9800
C16—H16C	0.9800	C42—H42C	0.9800
C17—C24	1.511 (8)	C43—C44	1.523 (8)
C17—C18	1.521 (8)	C43—C50	1.523 (8)
C17—H17A	1.0000	C43—H43	1.0000
C18—C23	1.368 (9)	C44—C49	1.376 (8)
C18—C19	1.379 (8)	C44—C45	1.381 (9)
C19—C20	1.380 (8)	C45—C46	1.383 (10)
C19—H19A	0.9500	C45—H45	0.9500
C20—C21	1.369 (9)	C46—C47	1.371 (9)
C20—H20A	0.9500	C46—H46	0.9500
C21—C22	1.352 (10)	C47—C48	1.3592
C21—H21A	0.9500	C47—H47	0.9500
C22—C23	1.372 (10)	C48—C49	1.377
C22—H22A	0.9500	C48—H48	0.9500
C23—H23	0.9500	C49—H49	0.9500
C24—H24A	0.9800	C50—H50A	0.9800
C24—H24B	0.9800	C50—H50B	0.9800
C24—H24C	0.9800	C50—H50C	0.9800
			
N6—Ni1—N3	179.51 (18)	N14—Ni2—N11	178.53 (18)
N6—Ni1—N2	95.11 (17)	N14—Ni2—N13	86.07 (17)
N3—Ni1—N2	85.24 (16)	N11—Ni2—N13	95.32 (18)
N6—Ni1—N5	85.33 (17)	N14—Ni2—N10	92.18 (17)
N3—Ni1—N5	94.32 (16)	N11—Ni2—N10	86.42 (17)
N2—Ni1—N5	178.97 (18)	N13—Ni2—N10	177.78 (18)
N6—Ni1—N8	91.56 (17)	N14—Ni2—N16	92.49 (18)
N3—Ni1—N8	88.08 (17)	N11—Ni2—N16	88.03 (17)
N2—Ni1—N8	92.18 (18)	N13—Ni2—N16	88.56 (18)
N5—Ni1—N8	88.74 (18)	N10—Ni2—N16	92.88 (18)
N6—Ni1—N7	88.78 (17)	N14—Ni2—N15	88.38 (18)
N3—Ni1—N7	91.58 (17)	N11—Ni2—N15	91.09 (19)
N2—Ni1—N7	87.14 (18)	N13—Ni2—N15	91.70 (19)
N5—Ni1—N7	91.94 (18)	N10—Ni2—N15	86.89 (19)
N8—Ni1—N7	179.3 (2)	N16—Ni2—N15	179.1 (2)
C1—N1—C8	115.7 (4)	C34—N9—C27	116.0 (5)
C1—N1—C9	116.7 (4)	C34—N9—C35	118.1 (4)
C8—N1—C9	117.1 (4)	C27—N9—C35	111.0 (4)
C1—N2—C2	115.8 (4)	C27—N10—C28	115.1 (4)
C1—N2—Ni1	113.2 (3)	C27—N10—Ni2	114.3 (3)
C2—N2—Ni1	105.1 (3)	C28—N10—Ni2	104.0 (3)
C1—N2—H2	107.5	C27—N10—H10	107.7
C2—N2—H2	107.5	C28—N10—H10	107.7
Ni1—N2—H2	107.5	Ni2—N10—H10	107.7
C3—N3—C4	113.8 (4)	C29—N11—C30	115.2 (5)
C3—N3—Ni1	106.7 (3)	C29—N11—Ni2	105.0 (3)
C4—N3—Ni1	112.6 (3)	C30—N11—Ni2	113.5 (3)
C3—N3—H3	107.8	C29—N11—H11	107.6
C4—N3—H3	107.8	C30—N11—H11	107.6
Ni1—N3—H3	107.8	Ni2—N11—H11	107.6
C4—N4—C5	118.4 (4)	C30—N12—C31	114.9 (5)
C4—N4—C17	119.4 (5)	C30—N12—C43	116.4 (5)
C5—N4—C17	119.2 (5)	C31—N12—C43	114.5 (5)
C6—N5—C5	114.9 (4)	C31—N13—C32	115.1 (4)
C6—N5—Ni1	105.2 (3)	C31—N13—Ni2	113.8 (3)
C5—N5—Ni1	113.6 (3)	C32—N13—Ni2	104.2 (3)
C6—N5—H5	107.6	C31—N13—H13	107.8
C5—N5—H5	107.6	C32—N13—H13	107.8
Ni1—N5—H5	107.6	Ni2—N13—H13	107.8
C7—N6—C8	116.5 (4)	C34—N14—C33	115.0 (4)
C7—N6—Ni1	105.4 (3)	C34—N14—Ni2	113.1 (3)
C8—N6—Ni1	112.8 (3)	C33—N14—Ni2	105.4 (3)
C7—N6—H6	107.2	C34—N14—H14	107.7
C8—N6—H6	107.2	C33—N14—H14	107.7
Ni1—N6—H6	107.2	Ni2—N14—H14	107.7
C25—N7—Ni1	160.6 (5)	C51—N15—Ni2	149.8 (5)
C26—N8—Ni1	166.6 (4)	C52—N16—Ni2	173.1 (5)
N1—C1—N2	108.7 (4)	N9—C27—N10	110.4 (4)
N1—C1—H1A	110.0	N9—C27—H27A	109.6
N2—C1—H1A	110.0	N10—C27—H27A	109.6
N1—C1—H1B	110.0	N9—C27—H27B	109.6
N2—C1—H1B	110.0	N10—C27—H27B	109.6
H1A—C1—H1B	108.3	H27A—C27—H27B	108.1
N2—C2—C3	109.0 (4)	N10—C28—C29	109.2 (4)
N2—C2—H2A	109.9	N10—C28—H28A	109.8
C3—C2—H2A	109.9	C29—C28—H28A	109.8
N2—C2—H2B	109.9	N10—C28—H28B	109.8
C3—C2—H2B	109.9	C29—C28—H28B	109.8
H2A—C2—H2B	108.3	H28A—C28—H28B	108.3
N3—C3—C2	108.7 (4)	N11—C29—C28	107.7 (4)
N3—C3—H3A	110.0	N11—C29—H29A	110.2
C2—C3—H3A	110.0	C28—C29—H29A	110.2
N3—C3—H3B	110.0	N11—C29—H29B	110.2
C2—C3—H3B	110.0	C28—C29—H29B	110.2
H3A—C3—H3B	108.3	H29A—C29—H29B	108.5
N4—C4—N3	114.0 (4)	N12—C30—N11	113.7 (5)
N4—C4—H4A	108.8	N12—C30—H30A	108.8
N3—C4—H4A	108.8	N11—C30—H30A	108.8
N4—C4—H4B	108.8	N12—C30—H30B	108.8
N3—C4—H4B	108.8	N11—C30—H30B	108.8
H4A—C4—H4B	107.6	H30A—C30—H30B	107.7
N4—C5—N5	114.3 (4)	N12—C31—N13	114.2 (5)
N4—C5—H5A	108.7	N12—C31—H31A	108.7
N5—C5—H5A	108.7	N13—C31—H31A	108.7
N4—C5—H5B	108.7	N12—C31—H31B	108.7
N5—C5—H5B	108.7	N13—C31—H31B	108.7
H5A—C5—H5B	107.6	H31A—C31—H31B	107.6
N5—C6—C7	108.1 (4)	N13—C32—C33	107.1 (4)
N5—C6—H6A	110.1	N13—C32—H32A	110.3
C7—C6—H6A	110.1	C33—C32—H32A	110.3
N5—C6—H6B	110.1	N13—C32—H32B	110.3
C7—C6—H6B	110.1	C33—C32—H32B	110.3
H6A—C6—H6B	108.4	H32A—C32—H32B	108.6
N6—C7—C6	109.1 (5)	N14—C33—C32	109.3 (5)
N6—C7—H7A	109.9	N14—C33—H33A	109.8
C6—C7—H7A	109.9	C32—C33—H33A	109.8
N6—C7—H7B	109.9	N14—C33—H33B	109.8
C6—C7—H7B	109.9	C32—C33—H33B	109.8
H7A—C7—H7B	108.3	H33A—C33—H33B	108.3
N1—C8—N6	107.9 (4)	N9—C34—N14	109.7 (5)
N1—C8—H8A	110.1	N9—C34—H34A	109.7
N6—C8—H8A	110.1	N14—C34—H34A	109.7
N1—C8—H8B	110.1	N9—C34—H34B	109.7
N6—C8—H8B	110.1	N14—C34—H34B	109.7
H8A—C8—H8B	108.4	H34A—C34—H34B	108.2
N1—C9—C10	108.7 (4)	N9—C35—C36	110.2 (5)
N1—C9—C16	112.6 (4)	N9—C35—C42	114.7 (5)
C10—C9—C16	114.3 (5)	C36—C35—C42	108.6 (4)
N1—C9—H9A	106.9	N9—C35—H35	107.7
C10—C9—H9A	106.9	C36—C35—H35	107.7
C16—C9—H9A	106.9	C42—C35—H35	107.7
C15—C10—C11	118.1 (5)	C37—C36—C41	119.1 (5)
C15—C10—C9	123.8 (5)	C37—C36—C35	119.4 (5)
C11—C10—C9	118.0 (5)	C41—C36—C35	121.5 (5)
C12—C11—C10	120.7 (5)	C36—C37—C38	120.3 (6)
C12—C11—H11A	119.7	C36—C37—H37	119.8
C10—C11—H11A	119.7	C38—C37—H37	119.8
C13—C12—C11	120.1 (6)	C39—C38—C37	119.8 (6)
C13—C12—H12A	120.0	C39—C38—H38	120.1
C11—C12—H12A	120.0	C37—C38—H38	120.1
C14—C13—C12	120.2 (6)	C38—C39—C40	120.6 (6)
C14—C13—H13A	119.9	C38—C39—H39	119.7
C12—C13—H13A	119.9	C40—C39—H39	119.7
C13—C14—C15	120.9 (6)	C39—C40—C41	120.0 (6)
C13—C14—H14A	119.5	C39—C40—H40	120.0
C15—C14—H14A	119.5	C41—C40—H40	120.0
C10—C15—C14	120.0 (5)	C40—C41—C36	120.1 (6)
C10—C15—H15A	120.0	C40—C41—H41	119.9
C14—C15—H15A	120.0	C36—C41—H41	119.9
C9—C16—H16A	109.5	C35—C42—H42A	109.5
C9—C16—H16B	109.5	C35—C42—H42B	109.5
H16A—C16—H16B	109.5	H42A—C42—H42B	109.5
C9—C16—H16C	109.5	C35—C42—H42C	109.5
H16A—C16—H16C	109.5	H42A—C42—H42C	109.5
H16B—C16—H16C	109.5	H42B—C42—H42C	109.5
N4—C17—C24	114.3 (5)	N12—C43—C44	111.2 (5)
N4—C17—C18	110.1 (4)	N12—C43—C50	111.5 (5)
C24—C17—C18	114.2 (5)	C44—C43—C50	107.4 (5)
N4—C17—H17A	105.8	N12—C43—H43	108.9
C24—C17—H17A	105.8	C44—C43—H43	108.9
C18—C17—H17A	105.8	C50—C43—H43	108.9
C23—C18—C19	117.2 (6)	C49—C44—C45	118.2 (6)
C23—C18—C17	123.1 (6)	C49—C44—C43	121.9 (6)
C19—C18—C17	119.7 (5)	C45—C44—C43	119.6 (6)
C18—C19—C20	121.0 (6)	C44—C45—C46	120.4 (6)
C18—C19—H19A	119.5	C44—C45—H45	119.8
C20—C19—H19A	119.5	C46—C45—H45	119.8
C21—C20—C19	120.6 (6)	C47—C46—C45	121.2 (7)
C21—C20—H20A	119.7	C47—C46—H46	119.4
C19—C20—H20A	119.7	C45—C46—H46	119.4
C22—C21—C20	118.4 (7)	C48—C47—C46	117.9
C22—C21—H21A	120.8	C48—C47—H47	121.1
C20—C21—H21A	120.8	C46—C47—H47	121.1
C21—C22—C23	121.2 (7)	C47—C48—C49	122.1
C21—C22—H22A	119.4	C47—C48—H48	119.0
C23—C22—H22A	119.4	C49—C48—H48	119.0
C18—C23—C22	121.6 (7)	C44—C49—C48	120.2
C18—C23—H23	119.2	C44—C49—H49	119.9
C22—C23—H23	119.2	C48—C49—H49	119.9
C17—C24—H24A	109.5	C43—C50—H50A	109.5
C17—C24—H24B	109.5	C43—C50—H50B	109.5
H24A—C24—H24B	109.5	H50A—C50—H50B	109.5
C17—C24—H24C	109.5	C43—C50—H50C	109.5
H24A—C24—H24C	109.5	H50A—C50—H50C	109.5
H24B—C24—H24C	109.5	H50B—C50—H50C	109.5
N7—C25—S1	178.6 (5)	N15—C51—S3	178.5 (6)
N8—C26—S2	179.0 (6)	N16—C52—S4	177.5 (5)
			
N6—Ni1—N2—C1	−36.9 (4)	C17—C18—C19—C20	−178.5 (6)
N3—Ni1—N2—C1	142.8 (3)	C17—C18—C23—C22	−179.6 (7)
N8—Ni1—N2—C1	54.9 (3)	N14—Ni2—N10—C27	40.1 (4)
N7—Ni1—N2—C1	−125.4 (4)	N11—Ni2—N10—C27	−140.3 (4)
N6—Ni1—N2—C2	−164.1 (3)	N16—Ni2—N10—C27	−52.5 (4)
N3—Ni1—N2—C2	15.5 (3)	N15—Ni2—N10—C27	128.4 (4)
N8—Ni1—N2—C2	−72.4 (3)	N14—Ni2—N10—C28	166.5 (3)
N7—Ni1—N2—C2	107.4 (3)	N11—Ni2—N10—C28	−14.0 (3)
N2—Ni1—N3—C3	14.0 (3)	N16—Ni2—N10—C28	73.9 (3)
N5—Ni1—N3—C3	−165.1 (3)	N15—Ni2—N10—C28	−105.3 (3)
N8—Ni1—N3—C3	106.3 (4)	N13—Ni2—N11—C29	162.0 (3)
N7—Ni1—N3—C3	−73.0 (3)	N10—Ni2—N11—C29	−16.6 (3)
N2—Ni1—N3—C4	139.6 (3)	N16—Ni2—N11—C29	−109.7 (4)
N5—Ni1—N3—C4	−39.5 (3)	N15—Ni2—N11—C29	70.2 (4)
N8—Ni1—N3—C4	−128.1 (3)	N13—Ni2—N11—C30	35.3 (4)
N7—Ni1—N3—C4	52.6 (3)	N10—Ni2—N11—C30	−143.3 (4)
N6—Ni1—N5—C6	−15.2 (3)	N16—Ni2—N11—C30	123.7 (4)
N3—Ni1—N5—C6	165.2 (3)	N15—Ni2—N11—C30	−56.5 (4)
N8—Ni1—N5—C6	−106.9 (3)	N14—Ni2—N13—C31	144.5 (4)
N7—Ni1—N5—C6	73.4 (4)	N11—Ni2—N13—C31	−35.0 (4)
N6—Ni1—N5—C5	−141.7 (4)	N16—Ni2—N13—C31	−122.9 (4)
N3—Ni1—N5—C5	38.6 (4)	N15—Ni2—N13—C31	56.2 (4)
N8—Ni1—N5—C5	126.6 (4)	N14—Ni2—N13—C32	18.3 (3)
N7—Ni1—N5—C5	−53.1 (4)	N11—Ni2—N13—C32	−161.2 (3)
N2—Ni1—N6—C7	166.0 (3)	N16—Ni2—N13—C32	110.9 (4)
N5—Ni1—N6—C7	−15.0 (3)	N15—Ni2—N13—C32	−69.9 (4)
N8—Ni1—N6—C7	73.6 (3)	N13—Ni2—N14—C34	138.9 (4)
N7—Ni1—N6—C7	−107.0 (4)	N10—Ni2—N14—C34	−42.4 (4)
N2—Ni1—N6—C8	37.9 (4)	N16—Ni2—N14—C34	50.5 (4)
N5—Ni1—N6—C8	−143.1 (4)	N15—Ni2—N14—C34	−129.3 (4)
N8—Ni1—N6—C8	−54.5 (4)	N13—Ni2—N14—C33	12.4 (3)
N7—Ni1—N6—C8	124.9 (4)	N10—Ni2—N14—C33	−168.9 (3)
N2—Ni1—N7—C25	25.3 (13)	N16—Ni2—N14—C33	−76.0 (3)
N5—Ni1—N7—C25	−155.2 (13)	N15—Ni2—N14—C33	104.2 (3)
N6—Ni1—N8—C26	−129.0 (18)	N14—Ni2—N15—C51	69.8 (9)
N3—Ni1—N8—C26	50.7 (18)	N11—Ni2—N15—C51	−108.9 (9)
N2—Ni1—N8—C26	135.8 (18)	N13—Ni2—N15—C51	155.8 (9)
N5—Ni1—N8—C26	−43.7 (18)	N10—Ni2—N15—C51	−22.5 (9)
C8—N1—C1—N2	−82.6 (6)	C34—N9—C27—N10	76.2 (6)
C9—N1—C1—N2	133.4 (5)	C35—N9—C27—N10	−145.2 (4)
C2—N2—C1—N1	177.8 (4)	C28—N10—C27—N9	−177.0 (4)
Ni1—N2—C1—N1	56.3 (5)	Ni2—N10—C27—N9	−56.7 (5)
C1—N2—C2—C3	−167.5 (4)	C27—N10—C28—C29	167.7 (4)
Ni1—N2—C2—C3	−41.8 (5)	Ni2—N10—C28—C29	41.9 (5)
C4—N3—C3—C2	−165.5 (4)	C30—N11—C29—C28	169.2 (4)
Ni1—N3—C3—C2	−40.6 (5)	Ni2—N11—C29—C28	43.6 (5)
N2—C2—C3—N3	57.1 (6)	N10—C28—C29—N11	−59.9 (5)
C5—N4—C4—N3	−71.6 (6)	C31—N12—C30—N11	74.2 (6)
C17—N4—C4—N3	88.8 (6)	C43—N12—C30—N11	−63.5 (7)
C3—N3—C4—N4	178.6 (4)	C29—N11—C30—N12	−177.1 (4)
Ni1—N3—C4—N4	56.9 (5)	Ni2—N11—C30—N12	−56.0 (6)
C4—N4—C5—N5	69.9 (6)	C30—N12—C31—N13	−73.8 (6)
C17—N4—C5—N5	−90.6 (6)	C43—N12—C31—N13	64.8 (6)
C6—N5—C5—N4	−175.2 (4)	C32—N13—C31—N12	174.8 (4)
Ni1—N5—C5—N4	−54.0 (5)	Ni2—N13—C31—N12	54.6 (6)
C5—N5—C6—C7	167.9 (4)	C31—N13—C32—C33	−170.1 (5)
Ni1—N5—C6—C7	42.2 (5)	Ni2—N13—C32—C33	−44.7 (5)
C8—N6—C7—C6	168.6 (4)	C34—N14—C33—C32	−166.4 (4)
Ni1—N6—C7—C6	42.7 (5)	Ni2—N14—C33—C32	−41.1 (5)
N5—C6—C7—N6	−59.0 (5)	N13—C32—C33—N14	59.9 (6)
C1—N1—C8—N6	83.6 (6)	C27—N9—C34—N14	−79.7 (6)
C9—N1—C8—N6	−132.5 (4)	C35—N9—C34—N14	144.8 (4)
C7—N6—C8—N1	179.5 (4)	C33—N14—C34—N9	−176.3 (4)
Ni1—N6—C8—N1	−58.4 (5)	Ni2—N14—C34—N9	62.5 (5)
C1—N1—C9—C10	−62.3 (6)	C34—N9—C35—C36	−145.4 (5)
C8—N1—C9—C10	154.2 (5)	C27—N9—C35—C36	77.0 (5)
C1—N1—C9—C16	65.5 (6)	C34—N9—C35—C42	−22.5 (7)
C8—N1—C9—C16	−78.0 (6)	C27—N9—C35—C42	−160.0 (5)
N1—C9—C10—C15	124.0 (5)	N9—C35—C36—C37	−145.6 (5)
C16—C9—C10—C15	−2.8 (8)	C42—C35—C36—C37	87.9 (6)
N1—C9—C10—C11	−55.3 (6)	N9—C35—C36—C41	37.5 (7)
C16—C9—C10—C11	177.9 (5)	C42—C35—C36—C41	−88.9 (6)
C15—C10—C11—C12	1.0 (8)	C35—C36—C37—C38	−175.5 (5)
C9—C10—C11—C12	−179.6 (5)	C36—C37—C38—C39	−0.5 (8)
C10—C11—C12—C13	−0.4 (8)	C37—C38—C39—C40	0.2 (8)
C11—C12—C13—C14	−0.2 (9)	C38—C39—C40—C41	−0.7 (9)
C12—C13—C14—C15	0.0 (9)	C39—C40—C41—C36	1.6 (8)
C11—C10—C15—C14	−1.2 (8)	C37—C36—C41—C40	−2.0 (8)
C9—C10—C15—C14	179.5 (5)	C35—C36—C41—C40	174.9 (5)
C13—C14—C15—C10	0.7 (9)	C30—N12—C43—C44	−162.1 (5)
C4—N4—C17—C24	−94.8 (6)	C31—N12—C43—C44	59.9 (6)
C5—N4—C17—C24	65.5 (7)	C30—N12—C43—C50	−42.4 (7)
C4—N4—C17—C18	135.1 (5)	C31—N12—C43—C50	179.7 (5)
C5—N4—C17—C18	−64.6 (7)	N12—C43—C44—C49	43.5 (8)
N4—C17—C18—C23	137.0 (6)	C50—C43—C44—C49	−78.6 (7)
C24—C17—C18—C23	6.8 (9)	N12—C43—C44—C45	−141.4 (6)
N4—C17—C18—C19	−43.7 (7)	C50—C43—C44—C45	96.5 (7)
C24—C17—C18—C19	−173.9 (5)	C43—C44—C45—C46	−173.5 (7)

### Hydrogen-bond geometry (Å, °)

**Table:**

*D*—H···*A*	*D*—H	H···*A*	*D*···*A*	*D*—H···*A*
N3—H3···S1^i^	0.93	2.82	3.439 (4)	125
N6—H6···S2^ii^	0.93	2.71	3.347 (4)	127
N11—H11···S3^ii^	0.93	2.89	3.614 (5)	136
N14—H14···S4^i^	0.93	2.66	3.418 (4)	139

Symmetry codes: (i) *x*−1, *y*, *z*; (ii) *x*+1, *y*, *z*.
